# Online audio-visual information on oral cancer for Spanish-speaking laypersons. A cross-sectional study

**DOI:** 10.4317/medoral.24770

**Published:** 2021-06-20

**Authors:** Yaima Ulloa-Morales, Francisco Negreira-Martínez, Andrés Blanco-Hortas, Berta Patiño-Castiñeira, Elena San-Román-Rodríguez, Pablo Varela-Centelles, Juan Manuel Seoane-Romero

**Affiliations:** 1 Department of Surgery and Medical-Surgical Specialities. School of Medicine and Dentistry. University of Santiago de Compostela. Spain; 2 Fundación IDIS. Lucus Augusti University Hospital. Galician Health Service. Lugo. Spain; 3 Service of Otorhinolaryngology. Lucus Augusti University Hospital. Galician Health Service. Lugo. Spain; 4 CS Praza do Ferrol. EOXI Lugo, A Mariña e Monforte. Galician Health Service. Lugo. Spain; 5 Department of Surgery and Medical-Surgical Specialities. School of Medicine and Health Sciences. University of Oviedo. Spain

## Abstract

**Background:**

Lack of knowledge and awareness of oral cancer seem to be the main causes of diagnostic delay. Online resources are often used by patients to obtain health/medical information. However, there are no reports on the quality and usefulness of oral cancer audio-visual resources in Spanish. The aims of this investigation were to disclose the type of information about oral cancer available, and whether it may be useful to shorten the patients’ oral cancer appraisal time-interval.

**Material and Methods:**

Cross-sectional study undertaken at three video-sharing sites on October, 13th 2019. Keywords: “Cáncer oral”; “cáncer de boca”. The first 100 results in each viewing list were retrieved by three reviewers. Demographical data was recorded, and interaction indexes, viewing rates, comprehensiveness, and usefulness were calculated for each video. The presence of non-scientifically supported information was also assessed. A descriptive analysis was undertaken, and relationships between variables were explored using the Spearman correlation test.

**Results:**

A total of 127 videos were selected. They were produced mainly by mass-media (46.5%; n=59) and their length ranged from 0.28 to 105.38 minutes (median 4.15 minutes; IQR: 2.34-9.67). The most viewed video (10,599,765 views; visualization rate 726,508.9) scored 0 both in usefulness and comprehensiveness. The most useful video gathered 44,119 views (visualization rate 2.033.13). A highly significant positive correlation (0.643; *p*<0.001) could be observed between usefulness and comprehensiveness of the videos, together with negative correlations between the visualization rate and usefulness (-0.186; *p*<0.05), and visualization rate and comprehensiveness (-0.183; *p*<0.05).

**Conclusions:**

Online audio-visual material about oral cancer in Spanish is incomplete, of limited usefulness, and often includes non-scientifically supported information. Most of these resources are produced by mass media and healthcare professionals, with minor contributions from educational and healthcare institutions. Visualization rates negatively correlated with the usefulness and comprehensiveness of the contents in these digital objects.

** Key words:**Oral cancer, diagnostic delay, patient education, internet, audio-visual resources, Spanish.

## Introduction

Oral cancer (OC) is estimated to account for 2% of all new cancer cases worldwide and for about the same proportion of neoplasms-related deaths. It is the most frequent cancer by incidence in Afghanistan, Papua-New Guinea, India, Pakistan, and Sri Lanka, and the most common type of cancer mortality for males in the latter three countries (1).

According to the latest data available from the International Agency for Research on Cancer, the worldwide projected age-standardised rate for this neoplasm is 4.0 cases per 100.000 inhabitants but in the geographical realm of Spanish language, oral cavity (and lip) cancer exhibits wide variations in incidence, ranging from 6.2 cases in the Caribbean region in 2018 to 1.4 in Central America. Female population experience a considerably lower incidence with the exception of Central America, where incidences are similar for both genders (1). Projections indicate important increments in incidence and mortality in the period 2020-2040, ranging from 32.1% and 36.6% for Spain to 118.5% and 122.2% for Equatorial Guinea.

Most OC cases are diagnosed at advanced stages (2), which is reported to have an impact on survival. In fact, survival to this neoplasm has not greatly improved for decades (3) despite the undeniable efforts of the scientific community. However, significant ameliorations may come from the side of early diagnosis, as survival rates may increase by about 30% if advanced OCs had been diagnosed at earlier stages (4), and diagnostic delay has proved to be a risk factor for advanced stage and mortality (5).

Among the many actors and processes influencing diagnostic delay, the patients’ appraisal time interval represents the major component of waiting times since the detection of a bodily change to the definitive diagnosis of OC (6). This phenomenon has been attributed to a general lack of knowledge and awareness of this disorder among the general public (7). This statement is particularly true for Spain, where 28% of the population had not even heard of OC (8) and 47% could not mention an OC-related sign or symptom, but also for many other Spanish-speaking populations (9-11).

This century has witnessed an enormous surge of the Internet, with a large proportion of the Spanish-speaking population accessing this network despite very wide regional differences. The ubiquitous presence of smartphones and similar mobile devices has made information readily available in a cost-free manner, and health-related information is not an exception. In fact, 75% of Internet users are reported to look for health/medical information and about 54% of patients with head and neck cancer rely on the Internet to find information about their treatment and collateral effects (12). The importance gained by online resources as suppliers of health-related information has raised concerns about the so-called “Dr Google” phenomenon and the quality of the information patients can obtain (13). In addition to quality, another worry about the use of online resources to disseminate health information is whether laypersons are able to understand it, as a certain level of literacy and reading comprehension is required. This proved to be a real barrier in the particular case of oral cancer-related websites (14,15). However, these difficulties disappear when the information is presented in an audio-visual format. In addition, dedicated online video-sharing sites have elicited enormous interest among social media users (16).

Unfortunately, most health-related videos lack validity for supporting the public in making health decisions (17). A recent study on the information about oral cancer available from YouTube® in English language unveiled a wide range of authors and contents with the most useful videos ranking late on the viewing list (18) and, therefore, with less chances to be viewed by the public. However, no reports on the quality and usefulness of Spanish-language audio-visual resources about oral cancer available through online public video repositories could be retrieved. Therefore, the aims of this investigation were to disclose the type of information about oral cancer are available through the main video-sharing online platforms, and whether the information they provide may be a useful contribution to shorten the patients’ appraisal time-interval in their path to a diagnosis of symptomatic oral cancer.

## Material and Methods

To achieve the aforementioned objectives, a cross-sectional study was designed, whose results are reported following the STROBE (STrengthening the Reporting of OBservational studies in Epidemiology) guidelines (19).

Audio-visual online information about oral cancer in Spanish was retrieved from the arguably three most popular video-sharing sites: YouTube® (www.youtube.com), Dailymotion® (www.daylimotion.com), and Vimeo® (www.vimeo.com) using the following key words: “cáncer de boca” and “cáncer oral”. The search was undertaken on October, 13th 2019 and the first 100 results in each viewing list (one search per key word per platform) were retrieved and their links copied into a spreadsheet.

Exclusion criteria included videos on oral cancer in animals, videos in languages other than Spanish, videos with no sound or headings, irrelevant videos (other topics or different types of cancer), advertisements, videos addressed to a specialized audience or presenting the findings of a research project.

Three researchers with different backgrounds analysed each clip of video: a final-year dental student (FN-M), a PhD student (YU-M), and a lecturer expert on oral cancer (JS-R). Demographical data (platform, title, publication date, length, number of views, and author) for each video were recorded as well as the interaction index suggested by Hassona *et al* (18) (number of likes – number of don’t likes, divided by the number of views and multiplied by 100) and the viewing rate (number of views, divided by the number of days since upload, and multiplied by 100). For the analysis of the contents of the films, six dimensions were considered (aetiology, risk factors, prevention, early detection, treatment, and prognosis). For a video to include a dimension, it should be expounded or, at least, mentioned. The usefulness of the contents of each video was assessed using a score system (18) that considers whether the video mentions the main risk factors for oral cancer (smoking, alcohol consumption, tobacco chewing, and HPV) allocating 1 point for each item. If the clip includes the main signs/symptoms of oral cancer (oral ulceration, colour change -white/red-, lump) receives another point per item mentioned. Additional points are allocated if representative images of oral cancer and/or potentially malignant disorders are included, and also when the video promotes prevention through early detection/avoidance of risk factors (18).

The presence of non-scientifically supported information was also assessed. Disagreements between reviewers were solved by consensus.

A descriptive analysis was undertaken, and results are presented as absolute and relative frequencies. The median was chosen as a central trend measure and the interquartile range as a spread indicator. Comparison between groups were undertaken using the Kruskal-Wallis test. The significance level chosen for the study was 5%. Relationships between variables were explored using the Spearman correlation test.

## Results

**Figure 1:**
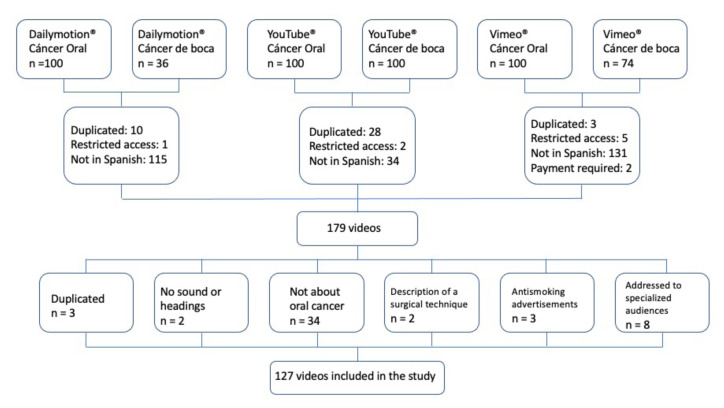
Flow chart of the study.

The YouTube® search permitted the retrieval of the intended 100 records per keyword, as occurred for Dailymotion® and Vimeo® for “cáncer oral”. Searches for “cáncer de boca” resulted in 74 hits in Vimeo® and 36 in Dailymotion.® The process of the selection of videos for the study is synthesized in Fig. 1.

Most of the 127 finally selected clips (Supplement 1) were retrieved from YouTube® (92.2%; n=117), with minor contributions from other repositories (DailyMotion®: 4.7% (n=6); Vimeo® 3.1% (n=4)). These videos were produced mainly by mass-media (46.5%; n=59), followed by individuals who identified themselves as healthcare professionals (21.2%; n=27), and laypersons (15.7%; n=20). Educational (7.9%; n=10) and healthcare (6.3%; n=8) institutions completed the sample, together with associations, enterprises, and other public institutions, each of them contributing with a single video.

Regarding their origin, most of them were published from Spain (29.92%; n=38), followed by Mexico (18.11%; n=23) and Chile (7.87%; n=10). Argentina and Colombia contributed with 9 videos each (7.09%), and Peru and the USA with 8. Creators from Ecuador and Paraguay uploaded another 5 and 3 videos respectively. Costa Rica and Panama contributed with 2 videos each, and El Salvador, Guatemala, Dominican Republic, Venezuela and the UK completed the list with one video each. The length of the clips included in the study ranged from 0.28 to 105.38 minutes (median 4.15 minutes; IQR: 2.34-9.67) and achieved a median visualization rate of 165.57 (IQR: 22.76-891.87).

The most viewed video (10,599,765 views; visualization rate 726,508.9) was 27.9 minutes long and it was uploaded to YouTube® by a US television channel, scoring 0 both in usefulness and comprehensiveness. The most useful video (10 points in usefulness) was uploaded by a Mexican healthcare professional and scored 5 in the 0-6 comprehensiveness scale and gathered 44,119 views (visualization rate 2,033.13).

Generally speaking, online OC videos in Spanish did not provide comprehensive information on oral cancer, with a median of two OC dimensions considered (IQR: 1.00-4.00) and a median usefulness score of 5.00 (IQR: 3.00-7.00). The interaction index (median 0.36; IQR: 0.19-0.74) was analysed only for those clips published in YouTube® because the other two repositories do not provide the information required for its calculation.

Despite being the most viewed group, those videos uploaded by laypersons resulted to be the less useful ones and the least comprehensive (Table 1) of all clips studied. The most useful videos resulted to be those authored by educational institutions, which also offered the widest perspective of the issue and a higher interaction index, despite being the less viewed only after those authored by mass media.

**Table 1:**
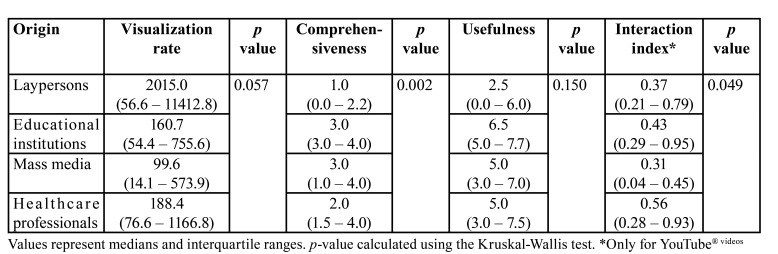
Scores by video origin.

Mass media videos were focused mainly on risk factors, particularly on tobacco smoking and alcohol consumption but included non-scientific information more frequently than other producers (*p*<0.001) (Table 2). Interestingly, laypersons-produced clips mention non-scientifically supported information less frequently than any other group and include more representative images than the largest uploader (mass media). The main strengths of the videos produced by educational institutions (the most useful in the study) were the inclusion of representative images (*p*=0.005), the mention of tobacco chewing (*p*=0.257), the inclusion of ulceration as a suspicious symptom (*p*=0.271), and the explicit recommendation for check-ups (*p*=0.263) and avoiding risk factors (*p*=0.160) (Table 2).

A highly significant positive correlation (0.643; *p*<0.001) could be observed between usefulness and comprehensiveness of the videos, together with negative correlations between the visualization rate and usefulness (-0.186; *p*<0.05), and visualization rate and comprehensiveness (-0.183; *p*<0.05).

**Table 2:**
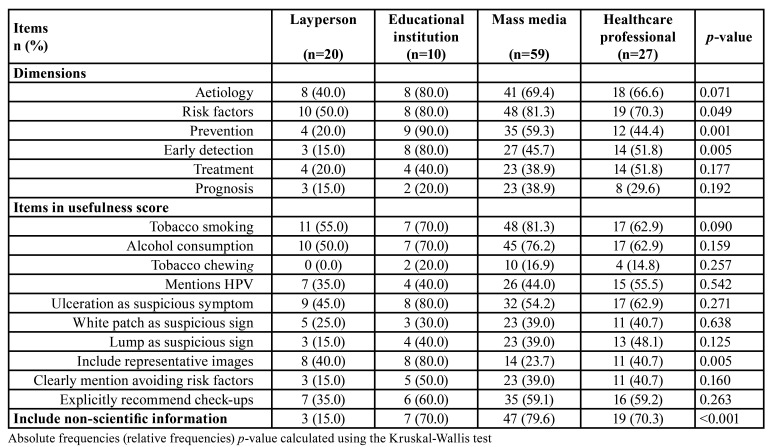
Items considered by video origin.

## Discussion

Public video repositories behave as social networks by sharing audio-visual contents, and the importance of these platforms becomes evident when considering that the most popular among them (YouTube®, San Bruno, CA, USA) has more than two billion registered users according to its own data. This privileged situation offers such a tremendous potential for health promotion and education and training that some scientific journals already run their own channels in these platforms (20). Conversely to what occurs with online written information (21), and to the best or our knowledge, there are no certifications or seals to endorse the quality of health-related audio-visual resources available on the Internet, with the subsequent risk for dissemination of misleading and unreliable information. In order to quantify this problem, the current study has focused on identifying the contents and categorise the usefulness of OC videos in Spanish language.

Our study has some limitations inherent to its cross-sectional design and the “snapshot” approach to data collection (18), which does not permit obtaining a sTable picture of the situation. In addition, the selected keywords -despite being among the most popular terms to describe this neoplasm- may also have conditioned the search results. On the other hand, this is the first investigation of OC videos in Spanish and its main strengths include the breadth of the search undertaken at three repositories and the participation of three reviewers with different backgrounds to ensure an adequate assessment of the variables studied.

Audio-visual information about OC available through the Internet in Spanish is usually incomplete: only a handful of creators managed to upload a comprehensive video about oral cancer, reaching the best performers median comprehensiveness scores of 3 (in a range 0-6). This information is of limited usefulness, and it can even be misleading in certain cases (Table 2). Besides, as most resources were produced by mass media (many are actually part of television programmes), it can be presumed that their main aim was other than to increase public’s knowledge of this neoplasm, which may explain their scores in the different items assessed in the current study. In addition, the source of videos that gathered most views (uploaded by laypersons) ranked the lowest in comprehensiveness and usefulness (Tables 1, Table 2) despite including non-scientifically supported information less frequently than their counterparts.

It is somehow surprising the relatively low scores attained by the group of clips produced by healthcare professionals, particularly in terms of usefulness (matching media produced clips) and comprehensiveness (lower than mass media’s), as they seem to be more focused than other creators on OC treatment, HPV, and OC warning signs (Table 2) while paying less attention to OC prevention and to less-known risk factors, such as smokeless tobacco. Furthermore, and according to our results, up to 70.3% of these videos include information not supported by scientific evidence. In these circumstances, it is worth questioning whether these authors are really healthcare professionals. It is in the nature of public video repositories on the Internet to allow free uploading of materials without checking the accuracy of their contents and the qualifications of their producers. In fact, this seems to be one of the reasons for their somehow astonishing success, but this strength easily turns into weakness when it comes to health-related information. This issue is even more relevant because this group of videos obtained the highest score in the interaction index, which may well have translated into individual exchange of inaccurate information with the audience that could well do more harm than good in some cases.

A similar study undertaken five years ago for English language resources about OC in the most popular repository (18) found that the most useful videos ranked late on the viewing list but failed to find a significant correlation between usefulness and viewing rate, which we could identify in the case of Spanish-language resources. Both studies agree on that clips produced by individual users were less useful than those produced by educational institutions and healthcare professionals.

The problem of the validity of health-related videos created for the public was addressed by Haslam *et al* (17) through an integrative review of papers reporting on studies about YouTube® videos on different health topics. They found that about one third of these papers allocated a good validity to this source of information for patients, while half of the studies recognised a poor validity of the clips studied. However, these platforms seem to be powerful instruments for patient education and action is needed from the Spanish-speaking oral health community to seize this means to deliver adequate and accurate messages to promote early diagnosis of oral cancer.

Since the implementation of quality seals for health-related information in public video repositories seems highly unlikely, it appears mandatory to guide prospective viewers to sound information by other means. Unfortunately, and according to our results, the self-identification as healthcare professionals is not sufficient and perhaps a better outcome may come from the creation of videos endorsed by professional boards, universities, and national health services. In addition, and considering both that relevant clips are consistently ranked late in visualization lists (18) and that the position of a given video in a visualization list is influenced by the number of views (which negatively correlates with its usefulness and comprehensiveness), efforts should be made to take advantage of current knowledge about the attributes that make videos highly accessible in public repositories namely (17) selecting adequate keywords, which may be obtained from reviewing existing popular videos; choosing short, attractive titles and using end cards; as well as exploiting creator’s networks for broad social sharing to gain “first-discovery advantage” to increase the likelihood of the video moving to a prominent place in the visualization lists. Also, promoting interaction with the audience, by opening the comments section and responding to viewers’ comments; and producing fast-paced videos or short videos, to keep viewers watching to the end are important issues. Additional points suggested by Haslam *et al* (17) include evoking emotions, as these videos are more frequently shared, as well as including storytelling, which makes videos more relaTable, sustains viewers’ interest and increases popularity. Re-uploading the video after certain time maintains the perception of relevance which, in combination with the supporting information for creators available from relevant Internet companies (17), would contribute to increase the impact of these contributions.

## Conclusions

Online audio-visual material about oral cancer in Spanish is incomplete, of limited usefulness, and often includes non-scientifically supported information. Most of these resources are produced by mass media and healthcare professionals, with minor contributions from educational and healthcare institutions. Visualization rates negatively correlated with the usefulness and comprehensiveness of the contents in these digital objects.
